# Heart Rate Variability and Cognitive Function: A Systematic Review

**DOI:** 10.3389/fnins.2019.00710

**Published:** 2019-07-09

**Authors:** Giuseppe Forte, Francesca Favieri, Maria Casagrande

**Affiliations:** ^1^Dipartimento di Psicologia, Sapienza Università di Roma, Rome, Italy; ^2^Dipartimento di Psicologia Dinamica e Clinica, Sapienza Università di Roma, Rome, Italy

**Keywords:** heart rate variability, global cognitive functioning, attention, executive functions, language, processing speed, memory, visuospatial skills

## Abstract

**Background:** Autonomic dysfunctions may precede the development of cognitive impairment, but the connection between these dimensions is unclear. This systematic review aims to analyze the relationship between heart rate variability (HRV) and cognitive functions.

**Methods:** The review process was conducted according to the PRISMA-Statement. Restrictions were made, selecting the studies in English and published in peer-review journals, including at least one cognitive measure and presenting the measurement of HRV. Studies that included participants with medical conditions, dementia, psychiatric disorders, strokes, and traumatic brain injury were excluded. Twenty studies were selected, with a total of 19,431 participants. The results were divided into different cognitive domains determined *a priori*: global cognitive functioning, attention, processing speed, executive functions, memory, language and visuospatial skills.

**Results:** Both increased sympathetic activity and decreased parasympathetic activity seem to be associated with a worse performance in the cognitive domains considered, in the absence of dementia and severe cardiovascular diseases or other medical and psychiatric diseases.

**Conclusion:** The results highlight the influence of the autonomic nervous system (ANS) in cognitive functioning. However, the marked interest facing toward a specific domain, i.e., the executive functions, and the relatively small number of the studies on this topic do not allow understanding better this relationship. Despite these limits, HRV could be considered a promising early biomarker of cognitive impairment in populations without dementia or stroke. This index should be evaluated within a preventative perspective to minimize the risk of developing cognitive impairment.

## Introduction

### Rationale

Cognitive functions are mental abilities that allow the correct interpretation and management of environmental information. These skills are distributed along a continuum that involves optimal cognitive functioning at one extreme and dementia at the other (Petersen, 2004). Proper cognitive functioning is essential to perform both the simplest tasks of everyday life and the most complex activities. Many factors can contribute to the physiological decline of cognitive functions in general, or of a specific domain, linked to the aging process (DeCarli, 2003; Murman, 2015).

Sometimes, the cognitive changes associated with aging became clinically significant and severe enough to compromise social and daily life functioning. The challenge of modern science, given the current sociodemographic conditions (i.e., population aging), is precisely to understand the reasons for this pathological decline, as well as to try to identify the early markers of cognitive impairment.

Cognitive functioning worsens under conditions of autonomic (Thayer and Lane, 2009; Thayer et al., 2010) and cardiovascular (O’donnell et al., 2012) dysfunctions. Within this perspective, a promising physiological correlate of cognitive functioning is heart rate variability (HRV) that is considered an index of autonomic control of the heart. HRV reflects the oscillations in the interval (ms) between consecutive heartbeats (R-R intervals) that result mainly from the dynamic interaction between the parasympathetic and the sympathetic inputs to the heart through the sinoatrial node (Malik, 1996; Thayer and Lane, 2000; Reyes del Paso et al., 2013).

Heart rate variability analysis can be conducted in the time domain, frequency domain, and by using non-linear analyses. In the time-domain, it is possible to calculate: (a) the standard deviation of all R–R intervals (SDNN) that reveals the components responsible for variability in the recording period (Malik, 1996); and (b) the root mean square of successive standard deviation (RMSSD) and the percentage of consecutive regular sinus RR intervals over 50 ms (pNN50) that should reflect vagal tone (Thayer and Lane, 2000; Kleiger et al., 2005; Shaffer et al., 2014; Laborde et al., 2017).

In the frequency domain, the oscillatory components are usually differentiated into different spectral profiles (Malik, 1996; Berntson et al., 1997; Reyes del Paso et al., 2013). Ultra-low frequencies (ULF; <0.0033 Hz) can only be evaluated using 24-h recordings, and reflect circadian oscillations, body temperature, metabolism, and activity of the renin-angiotensin system (Laborde et al., 2017). Very-low frequencies (VLF; 0.0033–0.04 Hz) represent long-term regulation mechanisms, thermoregulation, and hormonal mechanisms (Malik, 1996; Laborde et al., 2017). The low frequencies (LF; 0.04–0.15 Hz) reflect a mix between the sympathetic and vagal influences and are considered a marker of cardiac outflow influenced by both sympathetic and parasympathetic branches of the autonomic nervous system (ANS) (Malik, 1996; Laborde et al., 2017). Initially, it was assumed that only sympathetic outflow contributes to the LF-HRV. However, this view is not without controversial opinions. In particular, some authors suggest that LF-HRV primarily reflects parasympathetic influence (Reyes del Paso et al., 2013), and it is potentially affected by other cardiac mechanisms such as baroreflex sensitivity (e.g., Goldstein et al., 2011). High frequencies (HF; 0.15–0.40 Hz) reflect vagal tone (Malik, 1996; Laborde et al., 2017) and can be taken as an index of cardiac parasympathetic tone (Reyes del Paso et al., 2013). Finally, the LF/HF-HRV ratio has long been considered as an index of sympathovagal balance. However, this viewpoint has been strongly criticized (e.g., Billman et al., 2015), because the physiological bases are not clear (Laborde et al., 2017). For these reasons, this index, although widely used, would have a low predictive value (Laborde et al., 2017).

The cardiac vagal tone has frequently been linked to cognitive and emotional control (e.g., Porges, 1995; Hansen et al., 2003; Duschek et al., 2009). Within the HRV spectrum, the high-frequency band corresponds to parasympathetic cardiac activity. Parasympathetic influences are essential for the successful adaptation of the individual to changing environmental demands (Porges, 1995; Thayer and Lane, 2000, 2009; Reyes Del Paso et al., 2009). A reduction in vagal control (i.e., decreased HF-HRV) could indicate a lack of ability to respond flexibly to changing demands, reducing the range of possible options and thus limiting the individuals’ ability to generate appropriate responses and inhibit inappropriate ones.

According to the Neurovisceral Integration Model, there is an association between cardiac vagal tone and the functioning of attentional and emotional self-regulatory systems (Thayer and Lane, 2000, 2009). The neurovisceral integration hypothesis has suggested that the brain areas involved in self-regulation are also involved in cardiac autonomic activity through the vagus nerve (Ellis and Thayer, 2010; Thayer et al., 2012). These areas include the anterior, insular, and orbitofrontal cortices; amygdala; periaqueductal gray matter; ventral striatum; and autonomic motor nuclei of the brainstem (Thayer et al., 2012).

Further studies have confirmed the existence of an association between higher resting HRV and active inhibitory prefrontal-subcortical circuits (Thayer and Lane, 2000, 2009; Sakaki et al., 2016). In particular, higher resting-state HRV appears to be related to increased activity in executive brain regions (Thayer et al., 2012), while lower resting HRV seems to be related to hypoactive prefrontal regulation (Thayer and Sternberg, 2006; Park and Thayer, 2014). Consequently, a vagal control of the heart appear to be associated with the effective functioning of self-regulatory neural circuits, which permit the organism to respond quickly and flexibly to environmental demands (Thayer and Lane, 2000, 2009; Thayer and Friedman, 2004; Thayer et al., 2009; Thayer et al., 2012).

This hypothesis was formulated for the first-time considering emotion regulation and dysregulation (Thayer and Lane, 2000). According to this view, affective regulation requires selective attention to emotionally relevant stimuli and the inhibition of attention to irrelevant stimuli. Therefore, from a neurovisceral perspective, attentional and emotional regulations run together in the process of self-regulation and goal-directed behaviors. This extension of the neurovisceral hypothesis to other cognitive domains can allow improving the understanding of the relationship between the ANS and cognitive functioning.

### Aims

The general aims of this systematic review of the literature are: (a) to analyze the relationship between autonomic regulation and cognitive processes in the absence of affective dimensions and pathological aspects; (b) according to the hypothesis of the neurovisceral integration model, to understand the relationship between executive functioning and HRV; (c) to investigate the relationships between HRV and other cognitive domains (i.e., processing speed, attention, memory, language, visuospatial skills), to highlight whether HRV can be considered an index of general cognitive functioning; (d) to evaluate whether HRV can be considered as a predictor of cognitive performance.

## Methods

The review process was conducted according to the PRISMA-Statement (Liberati et al., 2009; Moher et al., 2009).

### Research Strategies

A systematic analysis of the international literature was carried out by selecting articles published in peer-review journals, using PubMed, PsycINFO, PsycARTICLES, and MEDLINE databases. The last research was conducted on June 10, 2018. Restrictions were made, limiting the study to academic publications in which the full text was published in English, and the study included human populations without age, gender, or ethnicity restrictions. The search strategy used the following syntax: “(cognit^*^ or neuropsych^*^) and (HRV or heart rate variability or vagal tone or vagal activity).”

### Eligibility Criteria

From the list of potential articles produced by systematic research, we selected the studies that included one or more cognitive measures and the measurement of HRV. Studies that included participants with medical conditions, which could potentially influence this relationship and those that included participants with a diagnosis of dementia, psychiatric disorders, strokes, and traumatic brain injury were excluded.

The first exclusion of non-inherent studies was made by analyzing titles and abstracts of the articles. Subsequently, the reading of the full text allowed further selection. Two researchers made the selection independently; inconsistent decisions between them were resolved by consulting a supervisor.

### Data Collection

According to the PICOS approach (Liberati et al., 2009), the following information has been extracted from each selected study: (1) author(s) and year of publication (2) characteristics of participants (including age, years of education, gender); (3) type of HRV measures (including measurement in the time or frequency domain); (4) cognitive domain analyzed (global, executive functions, processing speed, language, memory, attention, visuospatial skills); (5) nature and direction of the identified relationship. These data are summarized in Table 1. Only HRV resting measurements have been considered because the heterogeneity of cognitive tasks could influence recovery measures hindering finest comparisons between the variables.

**TABLE 1:** Participants’ characteristics, cognitive domains analyzed, HRV measurements, and links to cognitive performances in the selected studies.

**Study**	**Participants**				**Cognitive Domain**								
	**Group**	**N**	**Age M (SD)^a^**	**Sex (% men)^a^**	**GC**	**ME**	**EF**	**LG**	**AT**	**PS**	**VS**	**Domain HRV**	**Relation between HRV and cognitive performances**
Melis and Van Boxtel, 2001		52	22.0 (3.0)	48	✓							HF; MF^*^	Positive
Hansen et al., 2003		53	23.0				✓		✓			HF	Positive
Hansen et al., 2004		37	19.1				✓		✓			HF	Positive
Kim et al., 2006		311	65–85	0	✓							RMSSD; HF	Positive
Britton et al., 2008		5375	58.0 (6.0)	72	x	x	x	x	✓			SDNN; LF; HF.	No Relation
Duschek et al., 2009		60	24.5 (3.7)	47								MF^*^	Positive
Drucaroff et al., 2011		18	47.7 (15.7)	27.8			✓					SDNN; LF; HF	Positive
Shah et al., 2011		416	55.0 (2.9)		✓							HF	Positive
Solernó et al., 2012		19	21.5 (0.5)	47	✓						✓	RMSSD; SDNN; HF.	Positive
Frewen et al., 2013	Male Female	2145 2618	61.8 (8.3) 61.5 (8.39	100 0	✓	✓	x	✓	x		✓	SDNN; LF; LF/HF	Positive
Kimhy et al., 2013		817	57.11 (11.15)	44.2			✓					HF	Positive
Gillie et al., 2014		75	18.4	36.4		✓^b^						HF; LF	Positive
Zeki Al Hazzouri et al., 2014		869	76.0 (6.0)	41	✓			✓				SDNN; RMSSD	Positive
Mann et al., 2015		533	54.9 (10.7)	46.3			✓					HF	Positive
Williams et al., 2016		104	19.25 (1.43)	54					✓			HF	Positive
Mahinrad et al., 2016		3583	75.0 (3.0)	47	✓	x	✓			✓		HF	Positive
Colzato and Steenbergen, 2017	High HRV Low HRV	44 44	21.3 (0.3) 21.1 (0.3	43.2 43.2			✓	.				HF	Positive
Zeki Al Hazzouri et al., 2017		2118	45.0 (4.0)	42		x	✓					SDNN; RMSSD	Positive
Colzato et al., 2018		90	22.1 (2.5)	33.3			✓					RMSSD; HF	Positive
Ottaviani et al., 2018		50	24.2 (4.0)	38			✓					RMSSD; HF	Positive

M, mean; SD, standard deviation;, domain assessed but not resulted impairment in this study;, domain assessed and resulted impairment in this study; GC, global cognition; ME, memory; LG, language; AT, attention; EF, executive functioning; PS, information processing speed; VS, visuospatial skills; HF, high-frequency band; RMSSD, root mean square of successive RR interval differences; SDNN, standard deviation of NN intervals; LF, low-frequency band; LF/HF, ratio of LF-to-HF power; ^*a*^not reported in all studies; ^*b*^ability to suppress unwanted memory; ^*^MF, mid frequency band (0.06–0.14 Hz).

The neuropsychological tests used in the selected studies were associated, as defined by the authors, with some cognitive *a priori* domains (global functioning, attention, executive functions, memory, visuospatial skills, language, and processing speed). Performance in the various domains was analyzed, considering a single test or a composite score based on the measures of multiple neuropsychological tests (see Table 2).

**TABLE 2:** Neuropsychological tests used for the evaluation of the cognitive domains in the included studies.

**Cognitive domain**	**Task**	**Study**
Global cognition	Inductive reasoning tasks	Melis and Van Boxtel, 2001
	Mini-Mental State Examination (MMSE)	Kim et al., 2006; Mahinrad et al., 2016
	Modified Mini-Mental State Examination (3MSE)	Zeki Al Hazzouri et al., 2014
	Alice-Heim 4-I (AH4-I)	Britton et al., 2008
	Bennett–Seashore–Wesman Differential Aptitude Test	Solernó et al., 2012
	Montreal Cognitive Assessment (MoCA)	Frewen et al., 2013
Memory	Computerized Working Memory Test	Hansen et al., 2003, 2004
	20-word free recall test of short-term verbal memory	Britton et al., 2008
	Montreal Cognitive Assessment (MoCA) Subtest	Frewen et al., 2013
	Unwanted memory Test	Gillie et al., 2014
	Composite Score	Shah et al., 2011^a^
	Rey Auditory-Verbal Learning Test	Zeki Al Hazzouri et al., 2017
	Picture-Word Learning Test	Mahinrad et al., 2016
	Spanish and English verbal learning test (SEVLT)	Zeki Al Hazzouri et al., 2014
Language	Montreal Cognitive Assessment (MoCA) subtest	Frewen et al., 2013
	Mill Hill Vocabulary Test	Britton et al., 2008
Attention	Modified Flanker Task	Williams et al., 2016
	Montreal Cognitive Assessment (MoCA) Subtest	Frewen et al., 2013
	Test d2	Duschek et al., 2009
	California Computerized Assessment Package (CALCAP)	Hansen et al., 2003, 2004
Executive function	Montreal Cognitive Assessment (MoCA) Subtest	Frewen et al., 2013
	California Computerized Assessment Package (CALCAP)	Hansen et al., 2003, 2004
	Verbal fluency	Britton et al., 2008
	Computerized working memory task	Hansen et al., 2003, 2004
	Composite Score	Kimhy et al., 2013^b^; Mann et al., 2015^c^
	Stop-change paradigm	Colzato and Steenbergen, 2017
	Rule Shift Cards and the Hayling Sentence Completion Test	Ottaviani et al., 2018
	Iowa Gambling Task and Game of Dice Task	Drucaroff et al., 2011
Processing speed	Task-switching paradigm	Colzato et al., 2018
	Stroop Task	Mahinrad et al., 2016; Zeki Al Hazzouri et al., 2017
	Letter-Digit Coding	Mahinrad et al., 2016
Visuospatial abilities	Bennett–Seashore–Wesman Differential Aptitude Test	Solernó et al., 2012
	Montreal Cognitive Assessment (MoCA) Subtest	Frewen et al., 2013

*^*a*^Verbal Selective Reminding Test (SRT) and the visual SRT; ^*b*^Digits Backward task, Red/Green task (a variant of the classic Go/No Go) and Category Fluency; ^*c*^Brief Test of Adult Cognition (BTAC) and Stop and Go Switch Task (SGST).*

## Results

### Selection of the Studies

**FIGURE 1:**
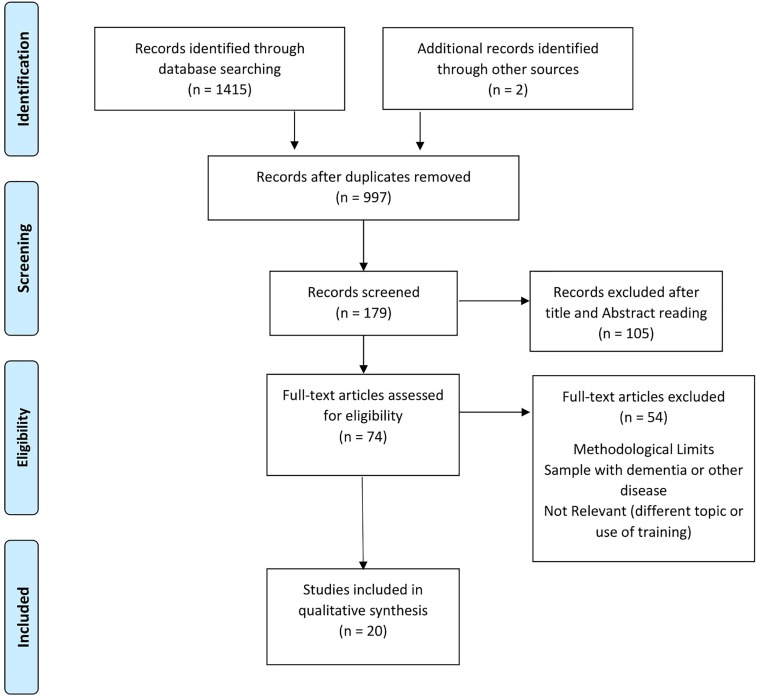
Studies selection flow diagram (PRISMA flow chart).

The flowchart shows the number of studies identified from the databases and examined by the authors, the number of articles, assessed for eligibility, and included in the review; the reasons for possible exclusions are also reported (Figure 1). The final analysis included 20 studies.

### Results of the Selected Studies

#### Demographic Data

The 20 studies that met the inclusion criteria were conducted from 2001 to 2018 and involved 19,431 people. Participants were aged between 18.4 (Gillie et al., 2014) and 76.0 years (Zeki Al Hazzouri et al., 2014). The percentage of men in the studies ranged from 0 (Kim et al., 2006) to 72% (Britton et al., 2008). Three studies did not report information about the participants’ gender (Hansen et al., 2003, 2004; Shah et al., 2011). Only one study presented a gender comparison (Frewen et al., 2013). Almost all of the researchers carried out a cross-sectional analysis, and only one study performed a longitudinal evaluation (Britton et al., 2008).

Many studies (Hansen et al., 2003, 2004; Kim et al., 2006; Britton et al., 2008; Frewen et al., 2013; Zeki Al Hazzouri et al., 2014; Mahinrad et al., 2016; Ottaviani et al., 2018) carried out statistical analysis controlling some confounding variables, such as demographics (age, gender, years of education, ethnicity), clinical (body mass index; blood pressure; heart rate; cardiovascular diseases, cholesterol, diabetes), and behavioral (smoking, exercise, alcohol consumption) variables.

### HRV Measurement

Except for one study (Mahinrad et al., 2016), HRV measurement was conducted by a continuous ECG recording, which lasted at least 5 min, as recommended by the guidelines of the European society of cardiology and the North American society (Malik, 1996).

Heart rate variability was evaluated considering time-domain analyses (Hansen et al., 2003; Zeki Al Hazzouri et al., 2014, 2017), frequency-domain analyses (Melis and Van Boxtel, 2001; Hansen et al., 2004; Duschek et al., 2009; Drucaroff et al., 2011; Kimhy et al., 2013; Gillie et al., 2014; Mann et al., 2015; Mahinrad et al., 2016; Williams et al., 2016; Colzato and Steenbergen, 2017; Colzato et al., 2018), or both (Kim et al., 2006; Britton et al., 2008; Drucaroff et al., 2011; Solernó et al., 2012; Frewen et al., 2013; Colzato et al., 2018; Ottaviani et al., 2018).

The HF-HRV analysis was the most frequently reported (Melis and Van Boxtel, 2001; Hansen et al., 2003, 2004; Kim et al., 2006; Britton et al., 2008; Drucaroff et al., 2011; Shah et al., 2011; Solernó et al., 2012; Kimhy et al., 2013; Gillie et al., 2014; Mann et al., 2015; Mahinrad et al., 2016; Williams et al., 2016; Colzato and Steenbergen, 2017; Colzato et al., 2018; Ottaviani et al., 2018). The LF/HF HRV ratio (Frewen et al., 2013), the LF-HRV band (Britton et al., 2008; Drucaroff et al., 2011; Frewen et al., 2013; Gillie et al., 2014), the mid-frequency (MF) HRV band (Melis and Van Boxtel, 2001; Duschek et al., 2009), the standard deviation of mean RR interval (SDNN) (Britton et al., 2008; Drucaroff et al., 2011; Solernó et al., 2012; Frewen et al., 2013; Zeki Al Hazzouri et al., 2014, 2017) and the square root of the mean squared differences of successive RR intervals (RMSSD) (Kim et al., 2006; Solernó et al., 2012; Zeki Al Hazzouri et al., 2014, 2017; Colzato et al., 2018; Ottaviani et al., 2018) were also evaluated.

### Cognitive Domain

All the cognitive domains were examined; global cognitive functioning (eight studies), memory (eight studies), language (two studies), attention (five studies), executive functions (thirteen studies), visuospatial skills (two studies), and processing speed (one study) (for references, see Table 1).

### HRV and Global Cognition (*n* = 8)

An association between HRV and global cognitive performance was reported. Only Britton et al. (2008) fail to find this relationship. Specifically, a low HRV was related to poorer performance (Melis and Van Boxtel, 2001; Kim et al., 2006; Solernó et al., 2012; Frewen et al., 2013; Zeki Al Hazzouri et al., 2014; Mahinrad et al., 2016) also after the adjustment of data for demographic, clinical and behavioral confounding variables (Kim et al., 2006; Frewen et al., 2013; Zeki Al Hazzouri et al., 2014; Mahinrad et al., 2016).

One study emphasized the role of the respiration rate (Melis and Van Boxtel, 2001), highlighting that poor reasoners had higher levels of sympathetic activity and respiratory rate than good reasoners.

### HRV and Memory (*n* = 8)

Three studies (Shah et al., 2011; Frewen et al., 2013; Gillie et al., 2014; Zeki Al Hazzouri et al., 2014) found a relationship between HRV and memory functionality, also after controlling demographic and clinical variables (Shah et al., 2011; Frewen et al., 2013).

People with higher HRV levels demonstrate a better ability to control over memory and a better ability to suppress unwanted memories (Gillie et al., 2014). Lower HRV is independently associated with a worse performance both in short and long-term verbal memory (Shah et al., 2011; Frewen et al., 2013).

However, some studies did not find a relationship between verbal (Britton et al., 2008; Mahinrad et al., 2016; Zeki Al Hazzouri et al., 2017) or visuospatial memory (Shah et al., 2011) and HRV.

### HRV and Language (*n* = 2)

Frewen et al. (2013) reported that reduced HRV is associated with lower linguistic performance, also after the adjustment of data for demographic, clinical and behavioral confounding variables. Conversely, Britton et al. (2008) did not find any relationship between HRV and linguistic performance.

### HRV and Attention (*n* = 5)

Four studies (Hansen et al., 2003, 2004; Duschek et al., 2009; Williams et al., 2016) found that individual differences in resting HRV predicted attentional performance. Lower HRV was associated with worse performance, also after the adjustment of the data for confounding variables (Duschek et al., 2009; Williams et al., 2016). However, these results were not confirmed by Frewen et al. (2013), who did not find any relationship between HRV and attention.

### HRV and Executive Functions (*n* = 13)

The executive domain was the most investigated. The studies demonstrated an association between lower HRV and poor executive performance; however, two studies (Britton et al., 2008; Frewen et al., 2013) did not confirm these findings.

Lower HRV predicted poorer performance on tasks involving executive functioning independently from demographic, clinical and behavioral confounding variables.

### HRV and Visuospatial Skills (*n* = 2)

Considering visuospatial abilities, only Frewen et al. (2013) observed a relationship with HRV. Lower HRV was associated with poor visuospatial performance, also after the adjustment of the data for demographic, clinical and behavioral confounding variables.

### HRV and Processing Speed (*n* = 1)

The only study (Mahinrad et al., 2016) that investigated processing speed showed that people with lower HRV had worse performance and experienced a higher decline in processing speed, independently from demographic and clinical characteristics.

## Discussion

### Summary of Evidence

The role of ANS on emotional regulation is well-known, whereas its links with cognitive functions are less well defined. Some studies concerning the general arousal (Lindsley, 1951), the attentional orienting (Sokolov, 1990), the alerting (Turpin and Siddle, 1978), and the regulation of actions (Jennings and van der Molen, 2005) have reported specific autonomic changes that were concurrent with cognitive functioning.

The first studies that have tried to identify a specific relationship between vagal tone and cognitive functions have highlighted changes in HRV depending on the type or complexity of the task (Lacey and Lacey, 1958; Richards and Casey, 1991). Based on these findings, some theories have been developed to explain the relationship between HRV and cognitive functioning. Among these, there is the Polyvagal Theory of Porges (1992), which highlights the importance of the vagus nerve for cognitive functions and in particular for the attentional processes. More recently, Thayer et al. (2009) developed the Neurovisceral Integration Model, which hypothesized a cortical integration between the executive, autonomic, and emotional functionality. The ANS is controlled by cortical circuits located in the prefrontal cortex, the anterior cingulate gyrus, the orbitofrontal cortex, and the amygdala, which are also crucial for cognitive and emotional processes (Critchley, 2009; Parasuraman and Jiang, 2012). The authors hypothesized that a sympathetic hyperactivation, with consequent prefrontal hypoactivation, would facilitate the disinhibition of the amygdala, i.e., an adaptive response; the amygdala would promote a decrease in HRV and an increase in heart rate (Thayer and Lane, 2009). This hypervigilant reaction would be related to reduced cognitive flexibility and vice versa; under parasympathetic activity conditions, the lack of prefrontal hypoactivation would be expressed through an increase in HRV with improved cognitive functions (Thayer and Lane, 2009).

One of the aims of this review was to analyze the neurovisceral integration hypothesis considering the performance that involved executive components in the absence of affective dimensions and pathological aspects.

A close examination of the selected studies confirms the relationships between resting HF-HRV and cognitive functioning, supporting the neurovisceral hypothesis in the absence of affective dimensions. The early results of Hansen et al. (2003) suggested a connection between resting HF-HRV, processing speed, and the accuracy of responses to monitoring tasks, with a stronger association when working memory was required; participants with high HF-HRV performed better than participants with low HF-HRV. The same set of tests was administered in a subsequent study (Hansen et al., 2004), and the results were replicated. These results were also confirmed during a condition of a threat of shock (Hansen et al., 2009).

Subsequently, numerous studies have analyzed the association between executive functions and HRV, considering both time and frequency domains, and in some cases with a large sample (Kimhy et al., 2013; Mahinrad et al., 2016; Zeki Al Hazzouri et al., 2017). A relationship with HRV was confirmed considering different executive functions (Hansen et al., 2004; Mann et al., 2015; Mahinrad et al., 2016; Colzato and Steenbergen, 2017). Moreover, participants with high resting-state HRV (indexed by RMSSD), as compared to participants with low resting-state HRV, demonstrated better action cascading (Colzato and Steenbergen, 2017), underlining that high resting HRV is associated with the optimal functioning of the prefrontal-subcortical inhibitory circuits that sustain flexible and adaptive responses to environmental demands (Colzato and Steenbergen, 2017).

Another aim of this review was to analyze the relationship between HRV and different cognitive domains. Many studies have found that reduced HRV in both time domain (RMSSD, SDNN) and frequency domain (HF, LF, LF/HF) were associated with weaker cognitive performance in both global cognition and specific cognitive domains.

Interestingly, the various HRV indices appear related to cognitive domains differently.

Lower LF-HRV, which is influenced by both sympathetic and parasympathetic branches of ANS, was linked to worse cognitive performance, in particular considering memory, language and global cognitive scores (Solernó et al., 2012; Frewen et al., 2013). Melis and Van Boxtel (2001) reported that high MF (Mid-Frequency band: 0.06–0.14 Hz), regulated by both the sympathetic and parasympathetic branches of the ANS, was associated with better performance in spatial tasks and to poorer verbal reasoning ability, while high HF-HRV was associated with better verbal reasoning ability. On the other hand, lower HF-HRV, which reflects vagal modulation, appears to be associated with weaker performance in global cognitive functions, such as those measured by the Mini-Mental State Examination (Kim et al., 2006), verbal reasoning abilities (Solernó et al., 2012), inhibition of memory responses (Gillie et al., 2014), or executive functions (Hansen et al., 2004; Mann et al., 2015; Mahinrad et al., 2016; Colzato and Steenbergen, 2017). These results can be due to the lateralisation of autonomic functions (Melis and Van Boxtel, 2001). In particular, sympathetic activation is related to visual and motor cortices, while parasympathetic activation is linked to the activity of prefrontal areas.

Moreover, some studies (Kim et al., 2006; Collins et al., 2012) reported a link between low HF-HRV and the risk of developing cognitive impairment. According to this hypothesis, low LF-HRV has been associated with white matter lesions in patients with Mild Cognitive Impairments (Zulli et al., 2005; Galluzzi et al., 2009) and Alzheimer’s Disease (Murakami et al., 2002; Zulli et al., 2005; de Vilhena Toledo and Junqueira, 2008). These results linked to others that identified a change in the LF/HF ratio based on the type and difficulty of the task (Luft et al., 2009; Mukherjee et al., 2011), reinforce the idea that the various parameters of HRV are associated with different cognitive functions.

The results under the umbrella of the memory domain are particularly fascinating. Although a general relationship between HRV and verbal memory was found, visual memory seems not to be associated with HRV. This finding can be explained by considering that many brain regions involved in visual functions, including parietal, temporal, and occipital lobes, all lie outside of the central autonomic network (Desimone, 1996; Pessoa et al., 2002). Therefore, HRV may correlate with verbal, but not visual, memory performance because verbal memory more specifically involves the central autonomic network.

In general, it is evident that executive functions, as well as global cognitive functioning, are the most investigated dimensions about HRV. The other cognitive domains (attention, processing speed, visuospatial skills, memory, and language) were the object of investigations that appear to be characterized by many methodological limits from both a quantitative and a qualitative point of view. A critical aspect of the studies measuring HRV is given by the numerous confounding variables. The results are particularly relevant when confounding variables are controlled; in some cases, the relationship became stronger, while in others, the adjustment for the confounding variables modifies the terms of the relationship. This pattern of results appears to indicate that other variables mediate the relationship between HRV and cognitive functions. HRV changes according to many factors, such as gender (Sztajzel et al., 2008), BMI (Koenig et al., 2014), anxiety (Chalmers et al., 2014), stress (Dishman et al., 2000), heart rate (Ga̧sior et al., 2016), and smoking habits (Levin et al., 1992; Karakaya et al., 2007), and so became important controlling these variables. Consequently, the analyses of their specific influence in mediating HRV effects on cognitive functioning are compelling. It is interesting to note that, in contrast to the other domains, the executive domain was significantly associated with HRV, above and beyond significant confounding variables (i.e., cardiovascular risk, age, and gender). This association is not surprising and reinforces the idea that HRV is strongly associated with the neuronal activity of the prefrontal cortex, which in turn regulates the executive functions (Thayer et al., 2009).

In contrast to results of other studies, Britton et al. (2008) did not show any correlation between HRV parameters and cognitive functioning, even if they considered a large cohort of people with characteristics similar to those of other studies. These inconsistent results could be due to the high variability of the data, which is attributable to some methodological procedures; for example, the participants were selected in different phases of one longitudinal study and this procedure can have implied effects due to both the survival and the selection of the sample. Another explanation could be the high percentage of males present in the sample (72%). Several studies show that men, compared to women, had a higher RR interval, a higher LF-HRV, and a lower HF-HRV (Koenig and Thayer, 2016). Finally, the tests used in this study (Whitehall II cognitive test battery) did not assess executive functions in detail.

Another aim of this study was to evaluate the predictive value of HRV for cognitive performance. The analyzed studies found that a higher HRV, both in time and frequency domains, were associated with finest cognitive performance, even after adjustment for the confounding variables commonly associated with HRV (i.e., age, gender, years of education, body mass index, blood pressure, cardiovascular diseases). Therefore, even if caution must be employed in defining the HRV as a predictor of performance in several cognitive domains, the results obtained from this review seem promising in that sense. However, more longitudinal studies and further research on poorly considered cognitive domains are needed to allow reliable inferences in this regard.

### Limitations

This systematic review of the literature aimed to carry out an analysis of the scientific studies concerning the link between the activity of the autonomic system and cognitive functioning.

Although we have tried to control the research methodology as much as possible, this study presents some limitations that could undermine the generalizability of the results. One weakness is given by the heterogeneity of the population and measures; this heterogeneity does not allow performing a quantitative analysis (i.e., meta-analysis) that would have given greater force to the conclusions.

Another limitation could be indirectly linked to the publication bias. The choice to include only academic articles published in peer-review journals may have limited the selection of only those studies that have obtained results in line with the literature. As a consequence, the results may overestimate this relationship. Moreover, the choice to select only studies published in English could have led to the elimination of studies conducted on other populations and written in different languages, further limiting the generalizability of the results. Moreover, the marked interest in a specific domain, i.e., the executive functions, and the relatively small number of studies in this topic does not allow to a conclusion concerning the involvement of the other cognitive domains. Finally, another limitation is represented by the overwhelming presence of cross-sectional studies that do not enable to few causal inferences on the relationship between HRV and cognitive functioning to be made.

### Future Perspectives

To overcome these limitations, in future psychophysiological studies it will be useful to utilize the emerging guidelines for reporting HRV parameters (e.g., Laborde et al., 2017), which can improve the quality of data, allow to more transparent reporting, and lead to more analysable data in quantitative analysis (e.g., meta-analysis).

Further research should aim to increase the studies on the relationship between HRV and some cognitive domains, such as attention, language, processing speed, and visuospatial skills., that are disregarded by the studies until now. Likely these cognitive domains have been neglected because they are never associated with an early cognitive impairment.

Of particular note is the attentional domain because it has been evaluated with tests that do not allow a complete assessment of this multidimensional construct.

Other essential aspects to consider in future studies are the vagal reactivity and the recovery processes that have been linked to cognitive performance (Capuana et al., 2014). Vagal reactivity represents the change between baseline and a specific event, like completing a task, and it is essential recording it to evaluate the individual’s adaptability to the situation (Laborde et al., 2018). Recovery is usually seen as a process of restoration; it refers to the change between the event and a time point after the event when the vagal activity has to be similar to the baseline. Comparable to vagal reactivity, vagal recovery plays a crucial role in the adaptability of the organism (Laborde et al., 2018). These two aspects are poorly analyzed with cognitive functioning. However, according to the vagal tank theory (Laborde et al., 2018), considering the vagal activity and the vagal recovery during different cognitive tasks could be interesting. This type of study could allow us understanding better how cardiac vagal control influences several key self-regulatory aspects of behavior and also evaluating whether the differences between baseline, task execution, and recovery are related to cognitive impairment.

## Conclusion

In this review, we focused on the analysis of the autonomic baseline that allows us to make inferences about the predictive value of autonomic homeostasis on cognitive impairment. Despite providing very relevant information, this analysis does not adequately enable the understanding of the mechanisms involved. Some studies that have analyzed HRV changes during the performance of cognitive tasks have shown that autonomic functionality varies according to the complexity and type of the task (Luft et al., 2009; Mukherjee et al., 2011).

Although this review has highlighted how some cognitive domains are more heavily investigated than others, in general, higher resting HRV is related to better performance in cognitive tasks. In contrast, lower resting HRV is associated with a lack of prefrontal control of the subcortical activity, which results in poor functioning of self-regulatory systems (Thayer and Lane, 2000; Thayer et al., 2009). In summary, a higher HF-HRV has been linked to better cognitive performance, and a lower HF-HRV has been associated with cognitive impairment.

In conclusion, this review highlights that the autonomous nervous system and the neurocognitive systems operate in close interaction. The results suggest that autonomic markers (LF, HF, LF/HF, SDNN) can be considered as early biomarkers for the measurement of cognitive impairment in populations without dementia or stroke. An initial analysis of these biomarkers could allow the implementation of preventative measures of autonomic control to prevent the worsening of cognitive decline.

## Author Contributions

MC and GF were responsible for the conception of the review, the literature research, and writing the manuscript. FF supervised the selection of the studies and contributed to the revision of the manuscript. All authors revised, read, and approved the submitted version.

## Conflict of Interest Statement

The authors declare that the research was conducted in the absence of any commercial or financial relationships that could be construed as a potential conflict of interest.
